# Associations of growth mindset, grit, and self-compassion with subjective vitality, physical education enjoyment, and exercise intention in university physical education: a complementary SEM and fsQCA analysis

**DOI:** 10.3389/fpsyg.2026.1914786

**Published:** 2026-10-30

**Authors:** Haoqun Yang, Wenzhou Shu, Shiwei Wang

**Affiliations:** 1 Applied Sport and Health Science, Lingnan University, Hong Kong, Hong Kong SAR, China; 2 School of French Studies, Sichuan International Studies University, Chongqing, China

**Keywords:** exercise intention, fuzzy-set qualitative comparative analysis, grit, growth mindset, physical education enjoyment, self-compassion, structural equation modelling, subjective vitality

## Abstract

**Inroduction:**

Drawing on Self-Determination Theory, Implicit Theories of Ability, and Self-Compassion Theory, this study examined cross-sectional associations of growth mindset, grit, and self-compassion with subjective vitality, physical education (PE) enjoyment, and exercise intention.

**Methods:**

Data from 1,500 undergraduates across eight Chinese universities were analysed using SEM and fsQCA.

**Results:**

In the revised partial-mediation model, growth mindset, grit, and self-compassion were positively associated with subjective vitality (β = 0.300, 0.221, 0.176) and PE enjoyment (β = 0.340, 0.137, 0.091). Growth mindset and grit also showed direct associations with exercise intention (β = 0.105, 0.137), whereas self-compassion did not (β = −0.032). Subjective vitality and PE enjoyment were positively associated with exercise intention (β = 0.220, 0.408), and the model explained 44.8% of its variance. Bias-corrected 95% CIs from 5,000 bootstrap samples excluded zero for all six specific indirect effects. Under the primary calibration scheme, fsQCA identified no single positive condition as necessary; the highest necessity consistency was 0.842 for growth mindset with high subjective vitality and 0.802 for grit with high PE enjoyment, both below 0.90. Two minimal sufficient configurations characterised high subjective vitality (GM*Grit and GM*SC; solution consistency = 0.810, coverage = 0.823), whereas no configuration met the sufficiency thresholds for high PE enjoyment.

**Discussion:**

The findings provide cross-sectional evidence consistent with the proposed framework but do not establish causal or temporal ordering.

## 1 Introduction

Recent pooled estimates indicate that insufficient activity among adults rose from 26.4% in 2010 to 31.3% in 2022 (Strain et al., 2024). The Chinese context presents a related concern. The Eighth National Survey on Students’ Constitution and Health documented improvement on several indicators while continuing to note weaknesses in the physical fitness of university students (Ministry of Education of the People’s Republic of China, 2021). University PE therefore offers an important setting for examining psychological factors associated with students’ engagement in physical activity.

Because PE participation is institutionally required at many Chinese universities, attendance alone says little about the quality or persistence of students’ engagement. Motivational accounts instead direct attention to personal resources that shape how students respond to challenge, effort, and setbacks. Three such resources are considered here. A growth mindset concerns viewing ability as developable (Dweck, 1999, 2006), grit concerns sustained interest and effort toward longer-term aims (Duckworth et al., 2007; Duckworth and Quinn, 2009), and self-compassion concerns responding to one’s own difficulties with understanding rather than harsh self-judgement (Neff, 2003). Each could support participation in sport and exercise, but they may do so in different ways.

Studying the three qualities in the same model raises several questions that remain open in PE research. One is comparative: when considered together, do they show similar or different associations with affective experience and exercise intention? A second concerns configuration. Standard SEM estimates average net relations, but it does not determine whether a condition must be present or whether different combinations can lead to the same outcome; those issues are central to configurational analysis (Furnari et al., 2021; Pappas and Woodside, 2021). A third issue is effect-size heterogeneity. Meta-analytic estimates for growth mindset and grit have generally been modest (Sisk et al., 2018, *r* ≈ 0.10; Credé et al., 2017, *r* ≈ 0.18), and the contrasting conclusions of Macnamara and Burgoyne (2023) and Burnette et al. (2023) further suggest that average effects may conceal important conditional patterns.

Against this background, the present study makes two substantive contributions. First, to our knowledge, it is the first to model three widely debated positive psychological qualities—growth mindset, grit, and self-compassion—jointly within a single sport/PE framework and to estimate their relative net associations with affective experience and exercise intention. Second, and more importantly, it adopts a configurational lens (fsQCA; Ragin, 2008) to examine whether these qualities operate as substitutes or complements, directly responding to Credé's (2018) call to study grit configurationally, and to examine configurational asymmetry. We do not claim methodological novelty for combining SEM and fsQCA: this integration has been advocated and applied previously (Woodside, 2013; Pappas and Woodside, 2021; Rasoolimanesh et al., 2021), including by Hao et al. (2025) in a comparable Chinese university PE context. Our aim is to bring this established dual-method design to bear on a set of constructs and a configurational question that have not previously been examined together.

Theoretically, the study proposes and examines an integrative dual-affective-association framework and uses a configurational perspective to characterise complementary and substitutable relationships among the three qualities. Practically, it provides an evidence base for differentiated cultivation of positive psychological qualities in university PE that can be tested in future longitudinal and intervention research.

## 2 Literature review and hypotheses

### 2.1 Self-determination theory and affective experiences in PE

Within SDT, subjective vitality refers to the felt energy available to the self and is closely tied to need satisfaction and internalised motivation (Ryan and Frederick, 1997; Ryan and Deci, 2001, 2017). PE enjoyment represents a related but more activity-specific positive response to participation (Kendzierski and DeCarlo, 1991). Evidence from PE research links autonomous motivation and positive affect with physical-activity outcomes (Vasconcellos et al., 2020), and longitudinal work has documented developmental change in motivation over time (Yun et al., 2025). We therefore examine vitality and PE enjoyment together as two distinct affective correlates within the proposed framework.

### 2.2 Growth mindset and sport engagement: the effect size debate and a conditional perspective

Dweck’s implicit theories of ability framework distinguishes entity beliefs from incremental beliefs about competence (Dweck and Leggett, 1988; Dweck, 1999, 2006). Biddle et al. (2003) developed the CNAAQ-2 to assess conceptions of athletic ability, and Wang et al. (2005) provided evidence of cross-cultural measurement equivalence.

Evidence concerning growth mindset is better characterised by heterogeneity than by a single agreed effect size. Sisk et al. (2018), drawing on a large meta-analytic database (Meta-Analysis 1: k = 273, *N* = 365,915), estimated an overall association of about *r* ≈ 0.10 but also reported meaningful subgroup variation. Later syntheses of interventions reached different conclusions. Macnamara and Burgoyne (2023), reviewing 122 studies, reported effects close to zero among studies meeting their best-practice criteria (*p* = 0.666) and raised concerns about possible conflicts of interest. Burnette et al. (2023), using 53 independent samples, found positive effects under particular targeting and implementation conditions (d = 0.14 for achievement and d = 0.32 for mental health), although their 95% prediction interval (−0.08 to 0.35) included zero.

Tipton et al. (2023) traced part of this disagreement to the way moderators were analysed: dichotomised moderator analyses and continuous multilevel meta-regression need not yield the same summary conclusion. Their reanalysis reinforced the importance of heterogeneity and conditionality. For the present study, the key implication is that growth-mindset associations may vary across people and contexts rather than appearing uniformly. Yan and Schuetze (2023) likewise argued that the processes connecting mindset beliefs with behaviour remain insufficiently specified and that interpretation of self-report measures may vary across cultural settings.

Sport-specific findings also point toward conditional patterns. McNeil et al. (2024) identified athlete profiles in which growth and fixed beliefs coexisted, with outcomes differing across profiles. Among Chinese athletes, Deng et al. (2025) reported indirect links between growth mindset and motivation through stress coping and basic psychological need satisfaction, while Zhang and Li (2025) found growth mindset to statistically mediate the association between sport atmosphere and psychological resilience in university students. These studies support examining mindset in relation to other psychological resources rather than treating it as an isolated attribute.

Accordingly, we hypothesise:

*H1a*: Growth mindset is positively associated with subjective vitality in university PE.

*H1b*: Growth mindset is positively associated with PE enjoyment in university PE.

### 2.3 Grit and sustained engagement: the construct uniqueness debate and a configurational turn

The Grit-S combines consistency of interest with perseverance of effort (Duckworth et al., 2007; Duckworth and Quinn, 2009), but treating these facets as a single construct remains debated. In a large meta-analysis (*N* = 66,807), Credé et al. (2017) reported substantial overlap between grit and conscientiousness (*ρ* = 0.84) and found stronger predictive performance for perseverance than for consistency of interest. Ponnock et al. (2020) likewise questioned the distinctiveness of grit from conscientiousness using multidimensional item-response modelling. Sport-specific evidence is more qualified: Cormier et al. (2024) found incremental predictive value for some performance and well-being outcomes after accounting for related traits, but not consistently across all outcomes.

Recent sport research also suggests that grit is not functionally uniform. Cormier et al. (2025) conceptualised it as a disposition that can develop through sport experience and may have both adaptive and costly consequences. Apró et al. (2024) reported different patterns for perseverance and consistency of interest, and Cocić et al. (2025) showed that the two facets were associated with different temporal patterns of practice accumulation. In the Chinese context, Zhang R. et al. (2023) developed the PE-Grit scale specifically for physical-education settings.

Credé (2018) explicitly recommended adopting a configurational perspective for studying grit, a recommendation that directly aligns with the fsQCA approach employed here. The association of grit with PE-related outcomes may depend on how it combines with other psychological qualities.

*H2a*: Grit is positively associated with subjective vitality in university PE.

*H2b*: Grit is positively associated with PE enjoyment in university PE.

### 2.4 Self-compassion in sport: measurement controversies and applications to physical activity

Self-compassion is typically described through self-kindness, common humanity, and mindfulness (Neff, 2003; Raes et al., 2011). In sport, early work with female athletes linked higher self-compassion with lower shame and fear of failure and showed that it explained variance beyond self-esteem (Mosewich et al., 2011, 2013).

How the Self-Compassion Scale should be scored is nevertheless disputed. Neff (2023) argues that a total score can represent an overall continuum of compassionate versus uncompassionate responding. Muris and Otgaar (2022), by contrast, caution that reversing negative self-responding items may mix protective and risk-related components in a single index. Because our primary questions concern self-compassion at the construct level, we retain the SCS-SF total score while making the scoring choice explicit and examining component-based sensitivity analyses.

Intervention and longitudinal studies provide additional evidence for its relevance to sport and activity. The RESET programme increased self-compassion among NCAA athletes, with larger gains among participants starting from lower baseline levels (Kuchar et al., 2023). In Chinese collegiate athletes, Zhang N. et al. (2023) linked self-compassion with emotional resilience after failure through vagal reactivity. Longitudinal evidence from Zhang et al. (2025) also connected self-compassion with later physical activity through barrier self-efficacy.

*H3a*: Self-compassion is positively associated with subjective vitality in university PE.

*H3b*: Self-compassion is positively associated with PE enjoyment in university PE.

### 2.5 Affective experience and exercise intention

As positive affective experiences, subjective vitality and PE enjoyment are expected to be positively associated with exercise intention (Hein et al., 2004).

*H4*: Subjective vitality is positively associated with exercise intention.

*H5*: PE enjoyment is positively associated with exercise intention.

*H6*: Growth mindset, grit, and self-compassion show positive statistical indirect associations with exercise intention through subjective vitality and PE enjoyment.

### 2.6 Research questions from a configurational perspective

Configurational research treats equifinality, conjunction, and asymmetry as features that may be missed by variable-centred net-effect models (Fiss, 2011; Woodside, 2013, 2014; Pappas and Woodside, 2021). Methodological work has therefore advocated using SEM-type approaches and fsQCA for different but complementary questions (Rasoolimanesh et al., 2021). This combination has already been applied in university PE research: Hao et al. (2025) examined learning motivation, and Jia et al. (2025) reported eight configurations associated with physical-exercise behaviour among Chinese female university students (consistency = 0.888, coverage = 0.722). Because necessity and sufficiency are set-theoretic properties rather than directional hypotheses, we address them through the following research questions:

**RQ1**: Are the condition combinations associated with high subjective vitality and high PE enjoyment unique, or do multiple equifinal sufficient configurations exist?

**RQ2**: Does any single positive psychological quality constitute a necessary condition for high affective experience?

**RQ3**: Are the configurations associated with non-high subjective vitality/PE enjoyment simple mirrors of those associated with high-level outcomes, or do they exhibit configurational asymmetry?

## 3 Methods

### 3.1 Research design

We used a cross-sectional survey and analysed the same dataset with two complementary approaches. SEM was used to estimate the average associations specified in H1–H6, whereas fsQCA examined whether combinations of conditions were linked to high or non-high affective experiences (RQ1–RQ3). All focal measures came from the same respondents at one measurement occasion. Accordingly, ‘direct effect’ and ‘indirect effect’ are retained only as SEM terminology and are not interpreted as evidence about temporal sequence or causal mediation (see Figure 1).

**Figure 1:**
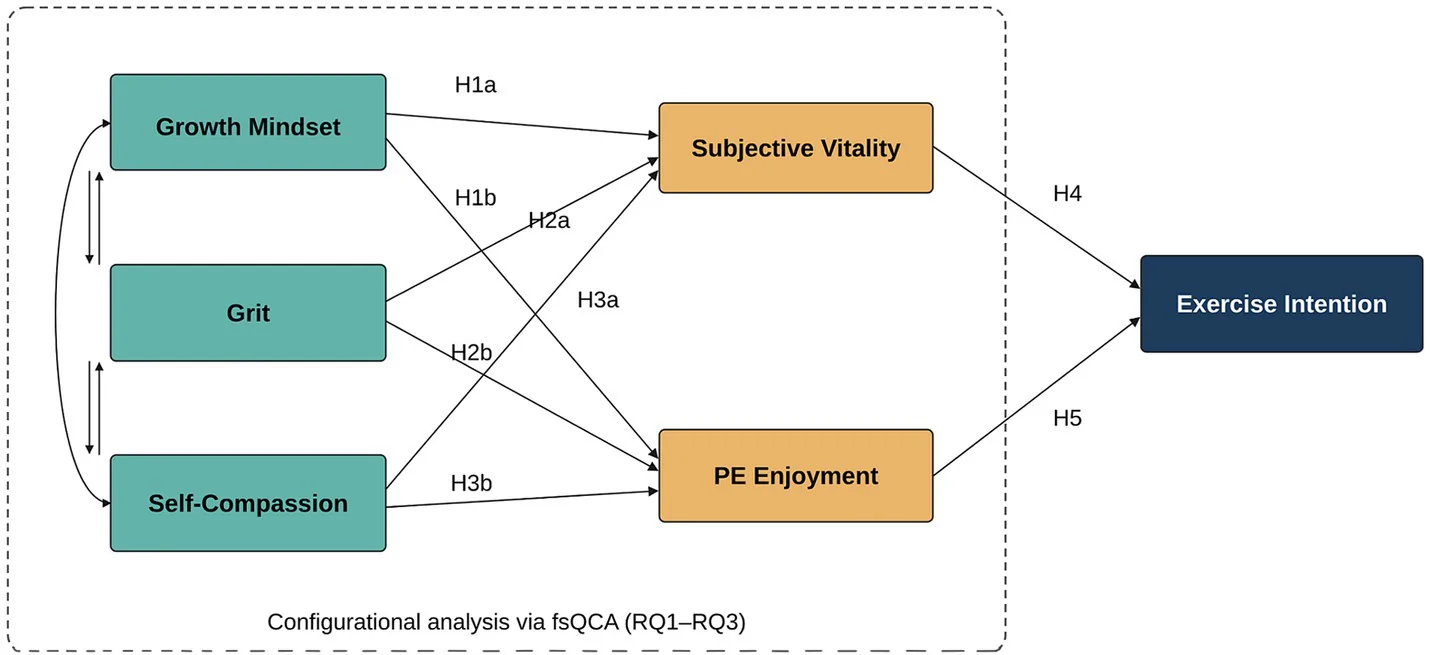
*A priori* conceptual framework of the study. Associations of growth mindset, grit, and self-compassion with subjective vitality and PE enjoyment correspond to H1–H3, and associations of the two affective experiences with exercise intention correspond to H4–H5. The dashed boundary indicates the scope of the configurational analysis via fsQCA (RQ1–RQ3). Double-headed arrows indicate that the three positive psychological qualities are allowed to correlate.

### 3.2 Participants, sampling, and data cleaning

Data collection took place from September to December 2025 through Wenjuanxing. Using convenience sampling, we invited undergraduates aged 18 years or older from eight universities in Guangdong, Jiangsu, and Sichuan: South China Normal University, Shenzhen University, and Jinan University in Guangdong Province; Nanjing Normal University, Southeast University, and Soochow University in Jiangsu Province; and Sichuan University and Southwest Jiaotong University in Sichuan Province. Of 1,583 submissions, 11 were removed because respondents were under 18, 18 because key scales were incomplete or more than 10% of responses were missing, 21 for failed attention checks, 16 for clear straight-line response patterns, and 17 for completion times under 180 s. The analytic sample therefore comprised 1,500 cases (94.8% of submissions), all complete on the core variables, so no additional imputation was applied.

The final sample comprised 759 males (50.6%) and 741 females (49.4%), with a mean age of 20.51 years (SD = 1.76). Freshmen through seniors accounted for 28.0%, 28.7%, 24.3%, and 19.0% of the sample, respectively. PE course types included ball sports (*n* = 463, 30.9%), fitness/conditioning (*n* = 475, 31.7%), swimming (*n* = 259, 17.3%), and other activities (*n* = 303, 20.2%). By university, the sample included 198 students from South China Normal University, 190 from Shenzhen University, 187 from Jinan University, 192 from Nanjing Normal University, 181 from Southeast University, 185 from Soochow University, 190 from Sichuan University, and 177 from Southwest Jiaotong University. Participants were drawn from 48 course classes taught by 24 instructors; robustness checks therefore accounted for clustering at both the university and class levels.

### 3.3 Chinese-language adaptation, pilot testing, and measures

Questionnaires were completed in Chinese. We used the published Chinese CNAAQ-2 (Wang et al., 2005) without retranslating it. For the other instruments, two bilingual Chinese-English researchers independently prepared forward translations and reconciled differences; a third bilingual expert, blind to the source wording, then back-translated the agreed version. Six experts reviewed semantic and cultural fit (I-CVI = 0.83–1.00; S-CVI/Ave = 0.96). Cognitive interviews with 12 undergraduates prompted minor changes to four items, after which the questionnaire was piloted with 60 students. The pilot median completion time was 9.3 min.

Growth mindset scores were calculated as the mean of the six incremental-belief items of the CNAAQ-2 (Biddle et al., 2003; Wang et al., 2005), each answered on a 5-point scale. The eight Grit-S items were also averaged (Duckworth and Quinn, 2009); its four consistency-of-interest items were reverse coded before computing the total. For the 12-item SCS-SF (Raes et al., 2011), self-judgement, isolation, and over-identification items were reversed and the item mean was used in line with Neff's (2023) total-score approach. Subjective vitality was represented by the mean of the seven SVS items on a 7-point scale, with Item 2 reversed (Ryan and Frederick, 1997). PE enjoyment used the eight-item PACES short form (Kendzierski and DeCarlo, 1991; Mullen et al., 2011), and exercise intention used four IPAS items (Hein et al., 2004); both instruments used 7-point response scales. Larger values on every composite denote higher standing on the named construct.

### 3.4 Common method bias controls

Several design and statistical checks were used to evaluate possible common-method influence. At administration, responses were anonymous, item order was randomised, and relevant instruments retained reverse-scored items. Analytically, we examined the first unrotated factor, fitted a one-factor CFA, and estimated an unmeasured latent method-factor model (Podsakoff et al., 2003). These checks were treated as diagnostics only; they cannot establish that common-method variance is absent.

### 3.5 Data analysis

SEM analyses were conducted in R with lavaan. The measurement model was examined before the structural specifications, consistent with Anderson and Gerbing's (1988) two-step approach. ML was the primary estimator with Likert indicators treated as approximately continuous; robustness checks re-estimated the model with MLR and with ordered-categorical WLSMV. We judged model fit using the pattern across χ^2^/df, CFI, TLI, RMSEA, and SRMR rather than any single index (Hu and Bentler, 1999; Kline, 2023). Reliability and validity were evaluated from Cronbach’s α, CR, McDonald’s ω, AVE, Fornell–Larcker comparisons (Fornell and Larcker, 1981), and HTMT.

Structural-model evaluation proceeded in stages. The original fully mediated specification was retained only as a constrained benchmark. A second model added GM → IN, Grit→IN, and SC → IN, and the final specification also estimated covariance between the residuals of concurrently measured SV and PE. With those four constraints released, the final model had the same degrees of freedom as the six-factor CFA; its global fit is therefore not presented as independent evidence for a causal structure. Direct, specific indirect, total indirect, and total effects were accompanied by bias-corrected 95% intervals from 5,000 bootstrap samples (Preacher and Hayes, 2008). Wald tests were used when comparing standardised path coefficients.

To address the nested structure arising from eight universities and 48 course classes, university-level ICC(1) values were first calculated. University-level cluster-robust inference used the CR2 bias-reduced linearisation estimator, also known as the Bell–McCaffrey small-sample correction (Bell and McCaffrey, 2002; Pustejovsky and Tipton, 2018). The eight universities were the clustering units. Statistical significance was assessed using two-sided t tests with coefficient-specific Satterthwaite degrees of freedom. A separate analysis using the 48 classes as clustering units served as a supplementary robustness check.

For fsQCA, fuzzy memberships were derived directly from the raw scale means. The 5th, 50th, and 95th percentiles served as the main anchors because no substantive membership cut-points had been specified in advance; they represented full non-membership, the crossover, and full membership. We also repeated the calibration under two alternatives to assess robustness. A condition was treated as necessary at consistency ≥ 0.90. Truth-table rows were coded as sufficient only when N ≥ 15, consistency ≥ 0.80, and PRI ≥ 0.65 (Mattke et al., 2021; Schneider and Wagemann, 2012). For reported configurations, we provide consistency, PRI, raw and unique coverage, and case frequency, and we compare complex, intermediate, and parsimonious solutions following Greckhamer et al. (2018). RoN was calculated as an additional check on the empirical relevance of candidate necessary conditions (Schneider and Wagemann, 2012). Sensitivity analyses recalibrated the data with 10th/50th/90th-percentile anchors and with mean −1 SD/median/mean +1 SD.

## 4 Results

### 4.1 Common method bias

The unrotated principal-component analysis produced six eigenvalues above 1, and its first component accounted for 34.8% of variance. Because that diagnostic is limited, we also compared CFA-based checks. The one-factor model fitted poorly (χ^2^/df = 8.74, CFI = 0.712, TLI = 0.695, RMSEA = 0.072, SRMR = 0.093), whereas the six-factor measurement model performed substantially better. In the ULMC specification, fit was χ^2^/df = 2.31, CFI = 0.960, TLI = 0.955, RMSEA = 0.030, and SRMR = 0.040; the method factor accounted for approximately 13.6% of variance. Controlling for that factor did not change which final-model paths were statistically significant, including the non-significant SC → IN path. These results suggest that method variance may be present but did not overturn the main inferential pattern.

### 4.2 Descriptive statistics and internal consistency

Descriptive statistics and internal-consistency estimates appear in Table 1. Across the six primary scales, α ranged from 0.794 to 0.906. At the facet level, the Grit-S estimates were lower: 0.665 for consistency of interest and 0.650 for perseverance of effort, both below the 0.70 rule of thumb cited by Hair et al. (2019). We therefore use the total Grit-S score in the primary analyses and reserve facet-specific findings for sensitivity checks.

**Table 1:** Descriptive statistics and internal consistency for the main variables (*N* = 1,500).

Variable	Items	M	SD	α	Skewness	Kurtosis
Growth mindset (GM)	6	3.85	0.72	0.891	−0.50	−0.15
Grit (total)	8	3.42	0.65	0.794	−0.21	−0.28
Consistency of interest (CI)	4	3.38	0.74	0.665	−0.15	−0.35
Perseverance of effort (PER)	4	3.46	0.68	0.650	−0.24	−0.26
Self-compassion (SC)	12	3.18	0.70	0.835	−0.14	−0.45
Subjective vitality (SV)	7	4.65	1.15	0.906	−0.21	−0.34
PE enjoyment (PE)	8	4.88	1.22	0.888	−0.40	−0.41
Exercise intention (IN)	4	4.72	1.30	0.843	−0.25	−0.50

GM, Grit, and SC were rated on 5-point scales; SV, PE, and IN were rated on 7-point scales.

### 4.3 Correlation analysis

Pearson correlations among the six core variables were all significant at *p* < 0.001. The three positive psychological qualities were moderately intercorrelated (*r* = 0.301–0.322), and each was positively correlated with subjective vitality, PE enjoyment, and exercise intention. PE enjoyment correlated more strongly with exercise intention (*r* = 0.604) than did subjective vitality (*r* = 0.532). These bivariate coefficients describe concurrent associations and are not interpreted as evidence of causal or temporal ordering (see Table 2).

**Table 2:** Correlation matrix for the main study variables (*N* = 1,500).

Variable	1	2	3	4	5	6
1. Growth mindset	1.000					
2. Grit	0.322***	1.000				
3. Self-compassion	0.301***	0.305***	1.000			
4. Subjective vitality	0.425***	0.372***	0.334***	1.000		
5. PE enjoyment	0.412***	0.274***	0.235***	0.556***	1.000	
6. Exercise intention	0.401***	0.355***	0.211***	0.532***	0.604***	1.000

***p < 0.001.

### 4.4 Confirmatory factor analysis and reliability/validity

For the six-factor measurement model, χ^2^ = 2446.0 (df = 930), giving χ^2^/df = 2.63; CFI = 0.953, TLI = 0.948, RMSEA = 0.033, and SRMR = 0.045. We interpreted this pattern as acceptable rather than relying on a single threshold. Standardised loadings ranged from 0.46 to 0.83 and were significant, with one Grit item and one SCS-SF item below 0.50. CR and ω exceeded 0.80 for every construct. AVE was above 0.50 for GM, SV, PE, and IN but was 0.443 for Grit and 0.459 for SC. Although Fornell–Larcker comparisons were satisfactory and all HTMT values were under 0.85 (maximum SV–PE = 0.63), the below-0.50 AVE values indicate borderline convergent validity for Grit and SC, which we retain as a limitation (see Table 3).

**Table 3:** Construct-level reliability and convergent validity.

Construct	α	AVE	CR	ω	√AVE
GM	0.891	0.575	0.890	0.890	0.758
Grit	0.794	0.443	0.862	0.862	0.665
SC	0.835	0.459	0.910	0.910	0.678
SV	0.906	0.586	0.908	0.908	0.766
PE	0.888	0.552	0.907	0.907	0.743
IN	0.843	0.610	0.862	0.862	0.781

AVE was below 0.50 for Grit and SC, although CR and ω were above 0.80; these constructs are therefore interpreted as showing borderline convergent validity.

### 4.5 Structural equation model and model comparison

Model comparisons are reported in Table 4. Adding the three predictor-to-intention paths improved fit relative to the full-mediation constraint (Δχ^2^ = 31.6, Δdf = 3, *p* < 0.001; ΔAIC = −25.6; ΔBIC = −9.7), so the fully mediated specification was not used as the primary model. Estimating the SV–PE residual covariance yielded an additional improvement (Δχ^2^ = 268.4, Δdf = 1, *p* < 0.001). The final structural component is saturated at the latent-variable level, whereas the overall model retains 930 degrees of freedom because of restrictions in the measurement component. Its global fit is therefore identical to that of the unrestricted six-factor CFA and cannot independently validate the hypothesised structural direction or distinguish it from covariance-equivalent alternatives (MacCallum et al., 1993).

**Table 4:** Comparison of structural equation models.

Model	χ^2^	df	χ^2^/df	CFI	TLI	RMSEA	SRMR	AIC	BIC
Full	2746.0	934	2.94	0.946	0.941	0.036	0.055	148732.6	149529.6
Partial	2714.4	931	2.92	0.947	0.942	0.036	0.052	148707.0	149519.9
Final (+SV ~ ~PE)	2446.0	930	2.63	0.953	0.948	0.033	0.045	148440.6	149258.8

Table 5 and Figure 2 summarise the standardised estimates from the final specification. Associations with SV were 0.300 for GM, 0.221 for Grit, and 0.176 for SC; the corresponding coefficients for PE enjoyment were 0.340, 0.137, and 0.091. Exercise intention retained direct associations with GM (β = 0.105) and Grit (β = 0.137), whereas SC → IN was not significant (β = −0.032, *p* = 0.131). The coefficients from SV and PE to intention were 0.220 and 0.408. Explained variance was 26.8% for SV, 19.9% for PE, and 44.8% for exercise intention.

**Table 5:** Standardised paths in the final partial-mediation model.

Path	β	SE	z	*p*	Result
GM → SV	0.300	0.024	12.51	<0.001	Significant
Grit → SV	0.221	0.024	9.20	<0.001	Significant
SC → SV	0.176	0.024	7.38	<0.001	Significant
GM → PE	0.340	0.025	13.55	<0.001	Significant
Grit → PE	0.137	0.025	5.45	<0.001	Significant
SC → PE	0.091	0.025	3.65	<0.001	Significant
GM → IN	0.105	0.022	4.66	<0.001	Significant
Grit → IN	0.137	0.021	6.39	<0.001	Significant
SC → IN	−0.032	0.021	−1.51	0.131	Not significant
SV → IN	0.220	0.025	8.83	<0.001	Significant
PE → IN	0.408	0.024	17.15	<0.001	Significant
SV ~ ~ PE residual correlation	0.432	—	—	<0.001	Significant

R^2^SV = 0.268; R^2^PE = 0.199; R^2^IN = 0.448. The final row reports the SV–PE residual correlation (r), rather than a regression coefficient (β). SE, z, and *p* refer to the primary ML analysis; cluster-robust inference is reported separately.

**Figure 2:**
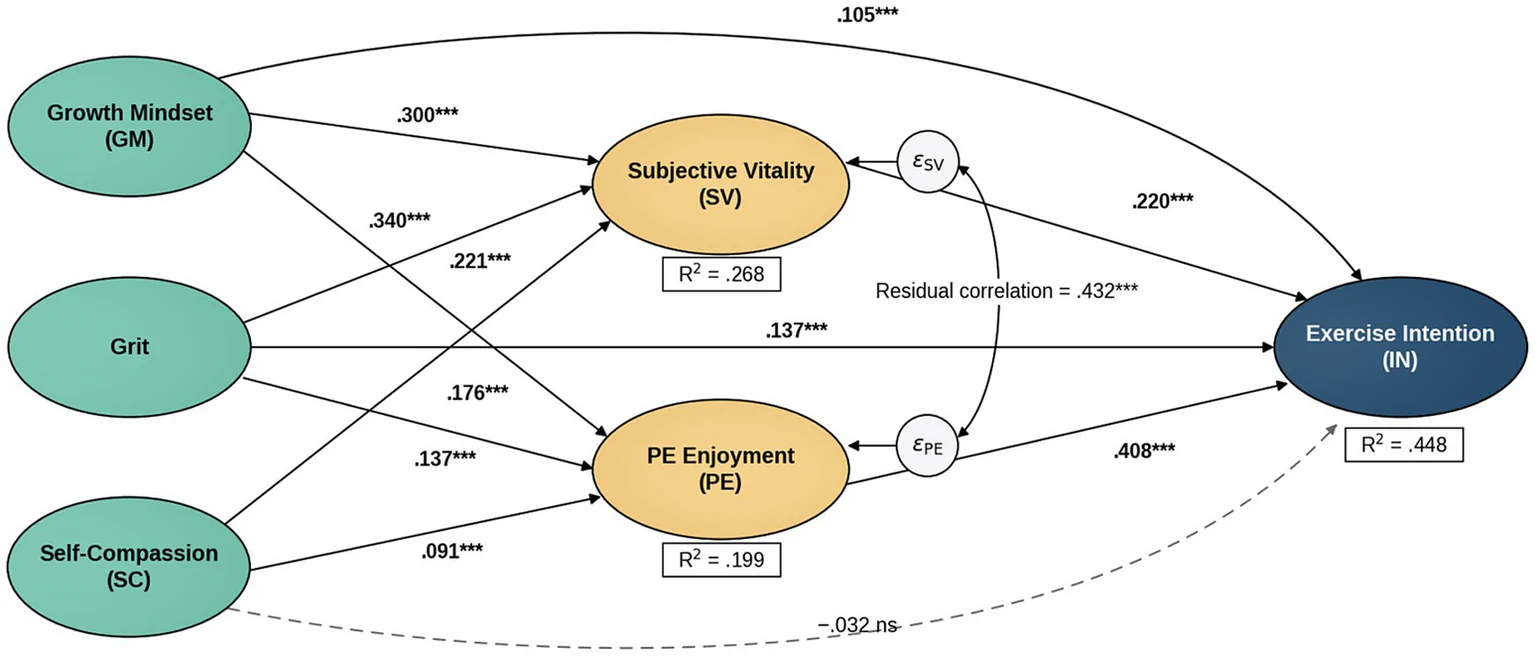
Standardised path coefficients for the final partial-mediation model (*N* = 1,500). Solid lines indicate statistically significant paths and the grey dashed line indicates a non-significant path; the residuals of SV and PE were allowed to correlate. R^2^ values are shown for each endogenous variable. GM, growth mindset; SC, self-compassion; SV, subjective vitality; PE, PE enjoyment; IN, exercise intention. ***p < 0.001; ns, non-significant.

Wald comparisons of the standardised coefficients showed that, in the subjective vitality equation, the GM coefficient was significantly larger than the Grit coefficient (difference = 0.079, *p* = 0.038) and the SC coefficient (difference = 0.124, *p* < 0.001), whereas Grit and SC did not differ significantly (*p* = 0.231). In the PE enjoyment equation, GM was likewise significantly larger than Grit (difference = 0.203, *p* < 0.001) and SC (difference = 0.249, *p* < 0.001), while Grit and SC again did not differ significantly (*p* = 0.241). Thus, growth mindset showed significantly stronger standardised associations with both affective experiences than the other two qualities, but the data do not support a statistically distinct Grit > SC ordering.

### 4.6 Direct, indirect, and total effects

Table 6 gives the bootstrap decomposition. For GM and Grit, both the direct paths to exercise intention and the specific indirect components through SV and PE had intervals that excluded zero. For SC, the direct-effect interval included zero, as did the interval for its total effect, but neither of its two specific indirect intervals did. Because the variables were observed concurrently, these quantities describe a cross-sectional statistical decomposition only and should not be read as evidence of causal mediation.

**Table 6:** Direct, indirect, and total effects in the final model (5,000 bootstrap samples).

Independent variable	Effect type	Estimate	BC 95% CI	CI includes 0
GM	Direct effect	0.105	[0.061, 0.147]	No
GM	Indirect via SV	0.066	[0.049, 0.086]	No
GM	Indirect via PE	0.139	[0.115, 0.164]	No
GM	Total indirect	0.205	[0.177, 0.235]	No
GM	Total effect	0.310	[0.264, 0.354]	No
Grit	Direct effect	0.137	[0.093, 0.182]	No
Grit	Indirect via SV	0.049	[0.036, 0.065]	No
Grit	Indirect via PE	0.056	[0.036, 0.077]	No
Grit	Total indirect	0.105	[0.078, 0.132]	No
Grit	Total effect	0.242	[0.192, 0.291]	No
SC	Direct effect	−0.032	[−0.074, 0.009]	Yes
SC	Indirect via SV	0.039	[0.027, 0.053]	No
SC	Indirect via PE	0.037	[0.017, 0.057]	No
SC	Total indirect	0.076	[0.049, 0.102]	No
SC	Total effect	0.044	[−0.003, 0.090]	Yes

### 4.7 Clustering, estimator, and construct-scoring sensitivity analyses

University-level ICC (1) values ranged from 0.002 to 0.007 across the six core variables, indicating little between-university variation. With university-cluster-robust standard errors, the six associations of GM, Grit, and SC with SV/PE remained significant; GM → IN, Grit→IN, SV → IN, and PE → IN also remained significant, whereas SC → IN remained non-significant. The same significance pattern was obtained when the 48 course classes were used as the clustering unit. Given that only eight university clusters were available, these analyses are treated as supplementary sensitivity checks on the standard errors rather than as multilevel causal models.

Estimator sensitivity analyses produced very similar overall fit under ML (CFI = 0.953, TLI = 0.948, RMSEA = 0.033, SRMR = 0.045), MLR (CFI = 0.952, TLI = 0.947, RMSEA = 0.033, SRMR = 0.044), and WLSMV (CFI = 0.955, TLI = 0.950, RMSEA = 0.032, SRMR = 0.043), indicating that the primary measurement structure was not sensitive to how the Likert items were treated.

Facet-based checks indicated that the aggregate scores concealed some heterogeneity. For Grit, consistency of interest was related to SV only weakly (β = 0.071, *p* = 0.014) and was not related to PE (β = 0.034, *p* = 0.257); perseverance of effort showed positive coefficients for both SV (β = 0.178) and PE (β = 0.116), with *p* < 0.001 in each case. For self-compassion, compassionate responding was positively related to SV (β = 0.106, *p* < 0.001) and PE (β = 0.071, *p* = 0.014). Uncompassionate responding showed a negative association with SV (β = −0.083, *p* = 0.002) and a non-significant negative coefficient for PE (β = −0.043, *p* = 0.125). We therefore retain total scores for comparability with prior work while treating the component results as evidence that the underlying facets need not behave identically.

### 4.8 Fuzzy-set qualitative comparative analysis (fsQCA)

The primary analysis used direct calibration at the 5th, 50th, and 95th percentiles (Table 7). Alternative calibration schemes used the 10th/50th/90th percentiles and mean −1 SD/median/mean +1 SD.

**Table 7:** Primary fsQCA calibration anchors.

Variable	Full non-membership (5th)	Crossover (50th)	Full membership (95th)
GM	2.56	3.97	4.93
Grit	2.31	3.47	4.45
SC	2.00	3.21	4.30
SV	2.69	4.73	6.47
PE	2.73	5.04	6.75
IN	2.49	4.83	6.77

Anchors are raw scale mean scores.

Under the primary calibration scheme, the necessity analysis showed that no single positive condition reached the 0.90 threshold (Table 8). For high SV, GM showed the highest consistency (0.842; coverage = 0.798; RoN = 0.824), followed by Grit (0.797; coverage = 0.759; RoN = 0.799) and SC (0.762; coverage = 0.746; RoN = 0.800). For high PE, Grit showed the highest consistency (0.802; coverage = 0.826; RoN = 0.847), followed by GM (0.767; coverage = 0.786; RoN = 0.816) and SC (0.753; coverage = 0.797; RoN = 0.834). Thus, none of the three positive psychological qualities can be described as a necessary condition in the set-theoretic sense.

**Table 8:** Necessity analysis and RoN for positive conditions.

Condition	Outcome	Consistency	Coverage	RoN	Necessary?
GM	High SV	0.842	0.798	0.824	No
GM	High PE	0.767	0.786	0.816	No
Grit	High SV	0.797	0.759	0.799	No
Grit	High PE	0.802	0.826	0.847	No
SC	High SV	0.762	0.746	0.800	No
SC	High PE	0.753	0.797	0.834	No

Necessity was assessed for positive conditions under the primary 5th/50th/95th-percentile calibration, using consistency ≥ 0.90 as the criterion.

Case frequencies for all eight logical combinations exceeded 15, so the truth table contained no logical remainders. Tables 9, 10 present the complete truth tables for high and non-high outcomes. For high SV, the rows 111, 110, and 101 met both the consistency and PRI thresholds. For high PE, no logical row simultaneously reached consistency ≥ 0.80 and PRI ≥ 0.65. For ~SV, rows 010, 001, and 000 met the sufficiency coding criteria, whereas ~PE did not yield a sufficient solution under the preset thresholds.

**Table 9:** fsQCA truth table for high subjective vitality and high PE enjoyment.

GM	Grit	SC	N	High SV Cons.	PRI	High SV code	High PE Cons.	PRI	High PE code
1	1	1	340	0.814	0.704	1	0.792	0.642	0
1	1	0	211	0.821	0.726	1	0.768	0.611	0
1	0	1	198	0.831	0.742	1	0.781	0.632	0
1	0	0	160	0.793	0.641	0	0.726	0.557	0
0	1	1	175	0.756	0.612	0	0.775	0.624	0
0	1	0	145	0.691	0.548	0	0.660	0.501	0
0	0	1	130	0.703	0.559	0	0.691	0.529	0
0	0	0	141	0.618	0.447	0	0.602	0.435	0

**Table 10:** fsQCA truth table for non-high subjective vitality and non-high PE enjoyment.

GM	Grit	SC	N	~SV Cons.	PRI	~SV code	~PE Cons.	PRI	~PE code
1	1	1	340	0.516	0.361	0	0.495	0.344	0
1	1	0	211	0.557	0.402	0	0.530	0.375	0
1	0	1	198	0.589	0.432	0	0.566	0.409	0
1	0	0	160	0.641	0.478	0	0.612	0.451	0
0	1	1	175	0.777	0.623	0	0.744	0.591	0
0	1	0	145	0.849	0.754	1	0.781	0.626	0
0	0	1	130	0.864	0.772	1	0.786	0.632	0
0	0	0	141	0.882	0.795	1	0.795	0.646	0

Truth-table coding thresholds were *N* ≥ 15, consistency ≥ 0.80, and PRI ≥ 0.65.

After Boolean minimisation, the minimal expression for high SV was GM*Grit + GM*SC. The GM*Grit* ~ SC term reported in the original manuscript is subsumed by the more general GM*Grit term and is therefore no longer presented as a separate configuration. Because there were no logical remainders, the complex, intermediate, and parsimonious solutions were identical; we therefore do not impose an additional core/peripheral distinction. The two sufficient configurations for high SV were GM*Grit (consistency = 0.803, PRI = 0.703) and GM*SC (consistency = 0.817, PRI = 0.716), with overall solution consistency = 0.810 and coverage = 0.823. For high PE, the three candidate combinations had consistency values of 0.769–0.784 and PRI values below 0.65, so none is interpreted as a sufficient configuration.

The minimal expression for non-high SV was ~GM* ~ Grit + ~ GM* ~ SC. The two sufficient configurations had consistency values of 0.870 and 0.854, with overall solution consistency = 0.860 and coverage = 0.521. The configurations for high and non-high SV contain sign-reversed condition terms, although the two solution expressions are not Boolean complements. This description does not imply a longitudinal or experimental causal mechanism (see Figure 3; Table 11).

**Figure 3:**
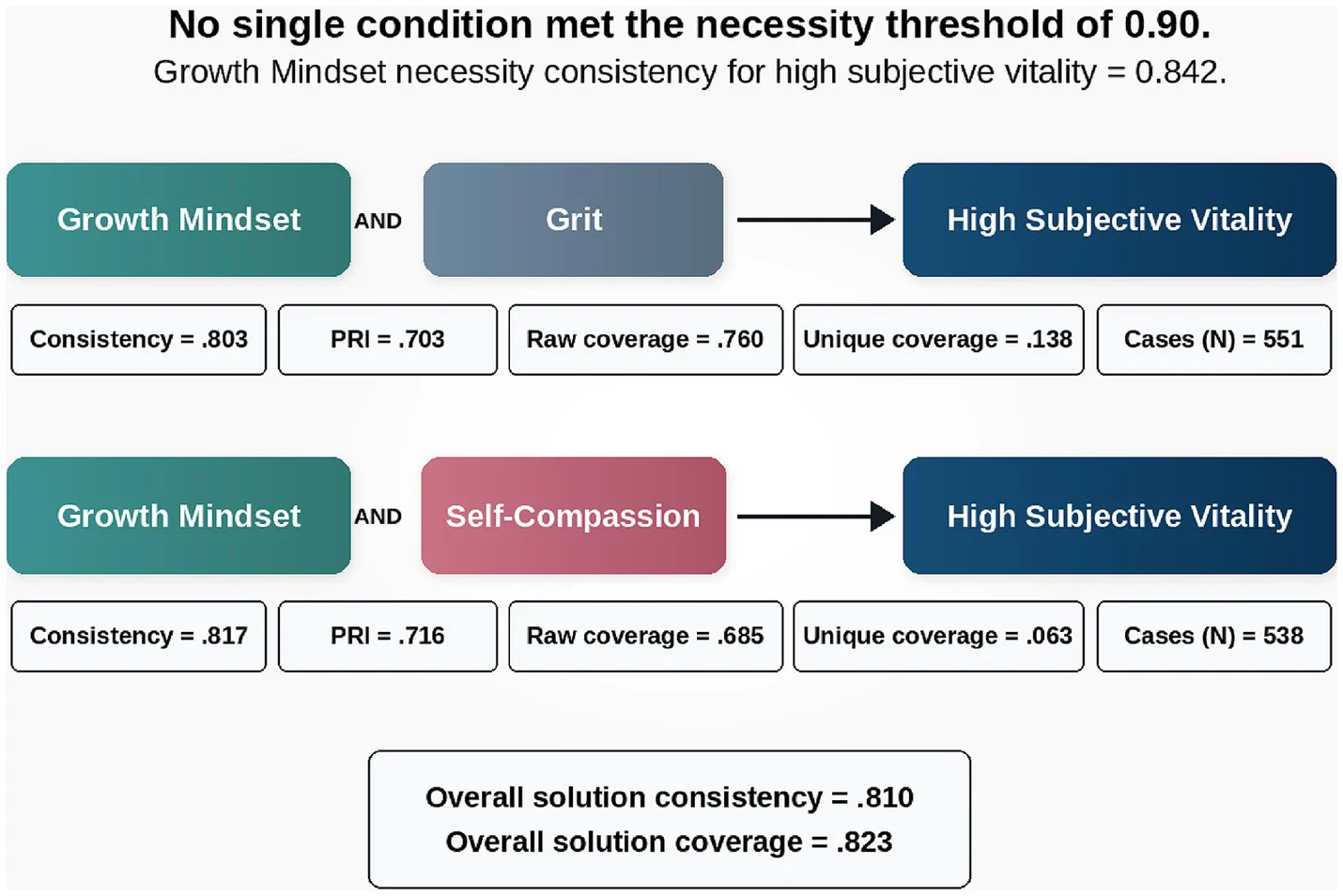
Two minimal sufficient configurations for high subjective vitality. No single positive condition reached the 0.90 necessity threshold under the primary calibration scheme; the necessity consistency of growth mindset for high subjective vitality was 0.842. The figure also reports consistency, PRI, coverage, and case frequency for both configurations.

**Table 11:** fsQCA sufficiency results.

Outcome	Configuration	Cons.	PRI	Raw cov.	Unique cov.	*N*	Status
High SV	GM*Grit	0.803	0.703	0.760	0.138	551	Sufficient
High SV	GM*SC	0.817	0.716	0.685	0.063	538	Sufficient
High PE	GM*SC	0.774	0.621	0.703	0.081	538	Not sufficient
High PE	GM*Grit	0.769	0.607	0.731	0.094	551	Not sufficient
High PE	Grit*SC	0.784	0.635	0.638	0.058	515	Not sufficient
~SV	~GM* ~ Grit	0.870	0.779	0.433	0.082	271	Sufficient
~SV	~GM* ~ SC	0.854	0.761	0.439	0.088	286	Sufficient
~PE	No sufficient solution	—	—	—	—	—	—

For high SV, overall solution consistency = 0.810 and coverage = 0.823; for ~SV, overall solution consistency = 0.860 and coverage = 0.521.

Calibration sensitivity analyses supported the sufficiency-based conclusions and configurational structure reported above (Table 12); necessity findings remain limited to the primary calibration scheme. Under both alternative calibration schemes, the consistency of GM*Grit and GM*SC for high SV remained above 0.80, while the highest candidate consistency for high PE remained below 0.80. Overall solution consistency and coverage for high SV and ~SV varied only slightly, with no sufficiency conclusion crossing a preset decision threshold.

**Table 12:** fsQCA calibration sensitivity analysis of sufficiency results.

Indicator	5/50/95	10/50/90	±1 SD/median	Interpretation
GM*Grit cons: High SV	0.803	0.812	0.806	All ≥ 0.80
GM*SC cons: High SV	0.817	0.824	0.820	All ≥ 0.80
High SV solution cons.	0.810	0.813	0.806	Stable
High SV solution cov.	0.823	0.811	0.818	Minor variation
Highest candidate cons: High PE	0.784	0.792	0.795	All < 0.80
~SV solution cons.	0.860	0.857	0.862	Stable
~SV solution cov.	0.521	0.514	0.528	Stable

## 5 Discussion

### 5.1 Net associations, model revision, and growth Mindset

The revised SEM no longer supports retaining the fully mediated model as the primary specification. Model fit improved when the three direct X → IN paths were added, and in the final model allowing residual covariance between SV and PE, GM and Grit each retained an independent direct association with exercise intention, whereas the direct association for SC was not significant. A fully mediated structure that includes only positive psychological qualities → affective experiences → exercise intention would therefore omit residual concurrent associations of GM and Grit with exercise intention. At the same time, PE enjoyment remained more strongly associated with exercise intention (β = 0.408) than did SV (β = 0.220), indicating that, within this sample and concurrent measurement design, PE enjoyment was more closely linked to exercise intention.

Across the two affective-experience equations, the standardised coefficients for GM were 0.300 and 0.340 and were significantly larger than those for Grit and SC in the Wald tests; the coefficients for Grit and SC did not differ significantly from one another. The data therefore support the comparative statement that GM showed the strongest association with both affective experiences, but they do not support a simple GM > Grit > SC ordering in which all pairwise differences are statistically distinct. These coefficients come from cross-sectional, single-source self-report data and are not directly comparable with effect sizes from growth-mindset intervention studies; nor can they answer whether changing growth mindset would lead to changes in affective experience or exercise intention.

### 5.2 Grit, self-compassion, and measurement debates

The Grit total score was positively associated with both SV and PE, but its two facets did not show the same pattern: perseverance of effort remained significantly associated with both outcomes, whereas consistency of interest was not significantly associated with PE. This is consistent with previous discussion of facet heterogeneity and the ‘jangle fallacy’ surrounding grit (Credé et al., 2017; Ponnock et al., 2020; Apró et al., 2024) and indicates that a total score provides a construct-level average rather than evidence that the constituent facets operate identically.

The standardised associations of the self-compassion total score with SV and PE were smaller, but the facet sensitivity analysis likewise revealed internal heterogeneity. Compassionate responding was positively associated with both affective experiences, whereas uncompassionate responding was negatively associated with SV and was not significantly associated with PE. Together with AVE values below 0.50 for Grit and SC, these results warrant a more cautious measurement interpretation: internal consistency and composite reliability were adequate, but convergent validity was borderline. Future work could more directly compare total-score, bifactor, and facet-level modelling strategies.

### 5.3 Interpreting the statistical indirect associations

Bootstrap results showed that the confidence intervals for all six specific indirect effects of GM, Grit, and SC through SV and PE excluded zero. GM and Grit each showed significant direct and indirect associations. For SC, the confidence intervals for the direct and total effects crossed zero, whereas both specific indirect effects were positive and excluded zero. This pattern suggests that the concurrent relationship between self-compassion and exercise intention may be expressed more through shared variation with positive affective experiences than through an independent direct association. Without temporal ordering or experimental manipulation, however, these ‘indirect effects’ can only be interpreted as a statistical decomposition consistent with the proposed mediation structure, not as proof of causal mediation.

The residual correlation between subjective vitality and PE enjoyment (r = 0.432) indicates a substantial association remaining after accounting for growth mindset, grit, and self-compassion. The three qualities explained 26.8% of vitality variance and 19.9% of enjoyment variance, suggesting that the proposed trait-based account captures only part of students’ affective experiences. Shared classroom experiences may contribute to the remaining association. For example, perceived competence, autonomy, relatedness, and teacher support may relate to both energy and pleasurable participation. Research in school PE links need support, need satisfaction, and intrinsic motivation with positive affect (Standage et al., 2005). These potential influences were not measured here.

Shared measurement influences may also contribute because both constructs were assessed through concurrent self-reports. Their empirical differentiation does not require their residuals to be uncorrelated, and the common-method diagnostics cannot exclude all shared-method variation. The residual association therefore neither establishes that the constructs are interchangeable nor indicates that one causes the other. It also cautions against treating the two indirect pathways as independent psychological mechanisms. Future research should assess classroom support, need satisfaction, and changing affective states through repeated measurements to examine whether these factors explain the shared variation.

### 5.4 Configurational perspective: two sufficient configurations for high and non-high vitality

The revised fsQCA results are more parsimonious than those reported in the original manuscript. First, no single positive psychological quality met the necessity threshold. For high SV, GM had the highest necessity consistency (0.842); for high PE, Grit had the highest (0.802). Both values, and all remaining positive-condition consistencies, were below 0.90, so none of the positive single conditions can be described as necessary under the primary calibration scheme. Second, Boolean minimisation yielded two sufficient configurations for high SV: GM*Grit and GM*SC. The GM*Grit* ~ SC term reported previously is subsumed by GM*Grit and should not be treated as a third independent route. Because all eight logical combinations contained sufficient cases, there were no logical remainders and the complex, intermediate, and parsimonious solutions were identical; accordingly, we do not assign additional substantive meaning to a core/peripheral distinction. A more precise interpretation is that GM appears in both minimal sufficient configurations, whereas Grit and SC form alternative conditions in combination with GM.

Third, ~SV was associated with two sufficient configurations, ~GM* ~ Grit and ~GM* ~ SC. Their condition terms are sign-reversed relative to the high-SV configurations, although the two expressions are not Boolean complements. These cross-sectional configurations do not imply that increasing a psychological quality will necessarily produce high vitality or that the absence of a quality will cause low vitality. Likewise, none of the candidate combinations for high PE simultaneously met the consistency and PRI thresholds, indicating that, in this sample and under the current calibration, the three psychological qualities did not form a stable sufficient configuration for high PE enjoyment. The calibration sensitivity analyses further showed that these sufficiency conclusions remained stable under two alternative calibration schemes.

### 5.5 Complementarity of SEM and fsQCA

The two analyses address different questions. SEM describes average net associations between each construct and the outcomes while controlling for the other variables and can quantify explained variance, direct associations, and statistical indirect associations. fsQCA instead evaluates whether set combinations of conditions meet prespecified criteria for necessity or sufficiency. In the present study, the two approaches are complementary: GM showed significantly stronger standardised associations with SV and PE than did Grit and SC in the SEM and also appeared in both minimal sufficient configurations for high SV, yet it did not meet the necessity criterion. Placing these results side by side makes clear that ‘a stronger average association’, ‘membership in sufficient configurations’, and ‘being a necessary condition’ are distinct propositions and should not be treated as interchangeable.

We do not treat the combination of SEM and fsQCA itself as a methodological innovation, because this complementary design has already been discussed and applied in prior work (Woodside, 2013; Pappas and Woodside, 2021; Rasoolimanesh et al., 2021; Hao et al., 2025). The contribution here lies in applying the design to three debated positive psychological qualities—growth mindset, grit, and self-compassion—and comparing their average associations and configurational relationships within the university PE context.

### 5.6 Potential practical implications

The practical implications of this study should be understood as hypotheses for future intervention research rather than as interventions already shown to be effective. First, the comparatively strong associations of GM with both positive affective experiences suggest that future studies could test whether combining beliefs about ability malleability with concrete motor-learning strategies, effort-focused feedback, and progress monitoring improves PE experiences. Second, the two sufficient configurations for high SV combine GM with either Grit or SC, suggesting that prospective research could compare ‘growth mindset + sustained effort’ and ‘growth mindset + self-compassion’ approaches and examine whether they benefit different students. Third, the strong concurrent association between PE enjoyment and exercise intention suggests that PE research may use enjoyment as an important outcome and test its temporal role in the development of sustained exercise intentions using longitudinal or experimental designs.

### 5.7 Limitations and future directions

Several limitations should be noted. First, the cross-sectional design cannot establish temporal ordering, and none of the direct, indirect, or configurational results should be interpreted as causal effects; future work should use longitudinal cross-lagged, intensive repeated-measures, or randomised intervention designs. Second, all core variables were self-reported at the same time point. Although Harman’s test, the single-factor CFA, and the ULMC model did not indicate that the main findings were fully accounted for by a common method factor, method bias cannot be ruled out. Future studies should incorporate behavioural data, teacher ratings, or objective activity indicators. Third, convenience sampling was used and the sample was limited to eight Chinese universities; generalisation to other cultures, age groups, and non-compulsory PE settings should therefore be cautious.

Fourth, AVE was below 0.50 for Grit and SC, and α was below 0.70 for both Grit facets, indicating that aspects of the measurement structure warrant further attention; the facet sensitivity analyses also showed that total scores can mask meaningful heterogeneity. Fifth, although university-level ICCs were low and university- and class-cluster-robust standard errors did not alter the primary inferential pattern, only eight university clusters were available, which precluded reliable estimation of a more complex multilevel structure. Sixth, fsQCA depends on calibration and threshold choices. We examined two alternative calibration schemes, but the sufficient configurations for high and non-high SV should be replicated in larger and more heterogeneous samples.

## 6 Conclusion

Using cross-sectional data from 1,500 Chinese university students, this study applied SEM and fsQCA as complementary approaches to examine relationships among growth mindset, grit, self-compassion, subjective vitality, PE enjoyment, and exercise intention in university PE. In the revised partial-mediation model, all three psychological qualities were positively associated with SV and PE, while GM and Grit also showed significant direct associations with exercise intention. Both SV and PE were positively associated with exercise intention, and the final model explained 44.8% of its variance. Bootstrap confidence intervals for all six specific indirect effects excluded zero, but these estimates represent statistical indirect associations and do not establish causal mediation.

Under the primary calibration scheme, fsQCA identified no single positive condition as necessary. High SV was characterised by two minimal sufficient configurations, GM*Grit and GM*SC, whereas no configuration for high PE met the preset sufficiency thresholds. The two sufficient configurations for ~SV (~GM* ~ Grit and ~GM* ~ SC) contained sign-reversed condition terms relative to the high-SV configurations, although the two solution expressions were not Boolean complements. Overall, the study provides cross-sectional evidence consistent with a Positive Psychological Qualities–Dual Affective Experience–Exercise Intention framework and shows how average net associations and set-theoretic configurations can describe psychological resource patterns in university PE from different perspectives.

## Data Availability

The raw data supporting the conclusions of this article will be made available by the authors, without undue reservation.
